# The Answer Bot Effect (ABE): A powerful new form of influence made possible by intelligent personal assistants and search engines

**DOI:** 10.1371/journal.pone.0268081

**Published:** 2022-06-01

**Authors:** Robert Epstein, Vivian Lee, Roger Mohr, Vanessa R. Zankich

**Affiliations:** American Institute for Behavioral Research and Technology, Vista, California, United States of America; National Institute of Technology Silchar, India, INDIA

## Abstract

We introduce and quantify a relatively new form of influence: the Answer Bot Effect (ABE). In a 2015 report in PNAS, researchers demonstrated the power that biased search results have to shift opinions and voting preferences without people’s knowledge–by up to 80% in some demographic groups. They labeled this phenomenon the Search Engine Manipulation Effect (SEME), speculating that its power derives from the high level of trust people have in algorithmically-generated content. We now describe three experiments with a total of 1,736 US participants conducted to determine to what extent giving users “the answer”–either via an answer box at the top of a page of search results or via a vocal reply to a question posed to an intelligent personal assistant (IPA)–might also impact opinions and votes. Participants were first given basic information about two candidates running for prime minister of Australia (this, in order to assure that participants were “undecided”), then asked questions about their voting preferences, then given answers to questions they posed about the candidates–either with answer boxes or with vocal answers on an Alexa simulator–and then asked again about their voting preferences. The experiments were controlled, randomized, double-blind, and counterbalanced. Experiments 1 and 2 demonstrated that answer boxes can shift voting preferences by as much as 38.6% and that the appearance of an answer box can reduce search times and clicks on search results. Experiment 3 demonstrated that even a single question-and-answer interaction on an IPA can shift voting preferences by more than 40%. Multiple questions posed to an IPA leading to answers that all have the same bias can shift voting preferences by more than 65%. Simple masking procedures still produced large opinion shifts while reducing awareness of bias to close to zero. ABE poses a serious threat to both democracy and human autonomy because (a) it produces large shifts in opinions and voting preferences with little or no user awareness, (b) it is an ephemeral form of influence that leaves no paper trail, and (c) worldwide, it is controlled almost exclusively by just four American tech companies. ABE will become a greater threat as people increasingly rely on IPAs for answers.

## 1. Introduction

### 1.1 Search results

Multiple studies conducted in recent years have demonstrated the power that search engines have to alter thinking and behavior by showing people biased search results [1–8, cf. 9–14], and research has also shown that these shifts can be produced without people’s awareness [2]. Bias in search results is difficult to see, and the few people who can spot it tend to shift their views even farther in the direction of the bias than people who cannot detect the bias [2, 15].

Search engines also influence people because of the trust people have in computer-generated output. Most people have no idea how search engines work [16–18] or, for that matter, how computers or algorithms work [19], and are oblivious to the various roles that humans play in generating computer output. Humans build the algorithms that computers use, for example, and those algorithms often produce biased content because of either the intentional or unconscious bias of the programmers [20–24]. Humans also modify existing programs–sometimes quite frequently. Recent reports suggest that Google’s ubiquitous search algorithm is manually adjusted more than 3,000 times a year, and those adjustments change both the content and the ordering of search results [25, 26]. Employees also deliberately add or delete content from blacklists and whitelists, which again has the effect of suppressing or boosting content [27–29]. People try to resist manipulation when they can see the human hand–authors’ names on news articles, guests on television and radio shows, videos on YouTube, and so on–but they think less critically when presented with algorithmic output, which they mistakenly believe to be inherently objective [30–34, cf. 35].

The human hand behind Big Tech companies is also invisible to users in another way. People are often oblivious to the many methods these companies are employing to collect personal data about them–the equivalent of more than three million pages of information about the average person who has been using the internet since its early days [36, cf. 37]. Monetizing that personal information is the bread and butter of Big Tech, which relies on the “surveillance business model” for nearly all its income [38–40]. Algorithms that match up users and vendors now direct the flow of hundreds of billions of dollars in purchases each year, but personal information can be used in other ways as well. As any con artist can tell you, the more you know about someone, the easier it is to manipulate him or her. Big Tech companies have accumulated massive databases about billions of people worldwide, and they are increasingly showing people personalized output that is optimized to draw clicks or impact a wide variety of thinking and behavior [15, 41–46, cf. 47, 48].

### 1.2 Search suggestions

Search results aren’t the only tools a search engine can wield to control people. Recent research shows that search suggestions–the short lists of words and phrases users are shown as they type characters into the search bar–can also shift thinking and behavior [15, 49, cf. 50–57]. Because negative (or “low-valence”) words draw far more attention and clicks than neutral or positive words [58, 59], one of the simplest ways to shift opinions to favor one candidate or cause is to suppress negative search terms for that candidate or cause. Google might have done so to support Hillary Clinton’s candidacy in the 2016 Presidential election [49, 60, 61, cf. 62].

### 1.3 Answer boxes

In 2014, Google began displaying boxes above their search results which contain a single answer to a person’s query, often accompanied by a link people can click to get more information [63]. Can these answers, now called “featured snippets” or “answer boxes,” also impact thinking and behavior? This is an important question not only because bias in a featured snippet might enhance the impact of biased search results and biased search suggestions, but also because an answer box could be considered a simple variant of a wide range of new content sources. Intelligent personal assistants (IPAs) such as Amazon’s Alexa, Apple’s Siri, Microsoft’s Cortana, and the Google Assistant (on Android devices and the Google Home device), all provide just one answer in response to a query. We are, in effect, moving away from search engines–platforms that provide thousands of possible answers in response to a query–toward the type of device we have seen portrayed in science fiction movies and television shows. On the original “Star Trek” episodes, when Captain Kirk wanted information, he didn’t consult a search engine; he simply said things like, “Computer, who’s the best looking captain in Star Fleet?” Why would one want a list of thousands of web pages when the computer can give you a simple answer?

Over time, Google–emulated to some extent by other, less popular search engines–has introduced several types of answer boxes, among them: a rich answer box (a type of featured snippet that includes additional information such as a graph, table, image, or interactive tool), a news stories box, a knowledge box (often information from Wikipedia displayed in the upper-right-hand corner of the search results page), a box suggesting related searches, and so on [64, 65]. Our focus, however, is on what Google calls the “featured snippet,” a relatively small box that is unlabeled and contains a simple answer to a user’s query [66]. On June 23, 2015, when people typed the query, “Who will be the next president?,” into the Google search bar, a featured snippet appeared reading, in part, “Hillary Clinton is the next President of the United States…. 10 Reasons Why Hillary Clinton Will Be the Next President” [67]. On October 22, 2017, when one of the authors of this paper typed “google play vs spotify” into the Google search bar, an answer box appeared immediately below the search bar reading, in part, “Google Play Music is my top pick after months of research and testing…. Google Play Music is better than Spotify–Business Insider” (S1 Fig). A link was included in the box to the relevant *Business Insider* article.

### 1.4 Answer bots and intelligent personal assistants

#### 1.4.1 An inevitable trend

For simplicity’s sake, we will refer to all electronic devices that provide simple answers to queries posed by humans as “answer bots” and define the Answer Bot Effect (ABE) as the extent to which answers provided by answer bots can alter people’s opinions and behaviors. It is important to measure this effect, we believe, because of what appears to be an inevitable trend: Worldwide, people are relying less and less on search results for their answers–just as, in the early 2000s, people began to rely less and less on books for their answers–and are simply accepting the answers they see in answer boxes or hear on their IPAs. Before answer boxes were introduced, people who used search engines had no choice but to click on search results and examine web pages to get their answers. As of 2016, approximately 43.9% of searches on mobile and desktop devices ended without a click; as of 2020, that percentage increased to 64.8% [68, 69; cf. 70]. Again, why click on a search result when the answer is right in front of you?

The shift toward answer bots is indicated by the increase in the number of people using IPAs. By 2019, there were 157 million smart speakers in American homes [71], and between 2019 and 2021, the number of Americans relying on voice assistants increased by nearly 20% [72]. Worldwide, more than 600 million smart speakers are expected to be in use by 2024 [72].

The spread of IPAs and answer boxes is not the only reason we need to measure and understand ABE. Children’s toys are increasingly internet-connected, and many of them answer children’s questions [73]. Hello Barbie has been around since 2015 and has been described as the perfect friend that can hold a two-way conversation and impact children’s attitudes about gender roles [74]. My Friend Cayla, a conversationally interactive toy released the same year was banned by the German government because of fears that hackers could intercept children’s questions and provide disturbing answers [75, 76, cf. 77]. Children are generally more impressionable than adults [78–80], which is why governments have often put restrictions on the kind of advertising that is directed toward young audiences [81]. With children’s toys answering questions–much of the time, with no parents around–both the questions children ask and the answers the toys provide can be inappropriate and potentially harmful [74, 82, cf. 83–85]. And, like search engines, these toys don’t just facilitate interactions; they also record them [86–88, cf. 89].

Both adults and children are also now conversing by the millions–sometimes knowingly, sometimes not–with chatbots, both through their computers and their mobile devices. When chatbots answer questions or promote viewpoints, they too can shift opinions and behavior [90, cf. 91]. The number of people currently conversing with chatbots is difficult to estimate, but it is certainly a large number that is increasing rapidly [92, 93]. When dating website Ashley Madison was hacked in 2015, the hackers learned, among other things, that “20 million men out of 31 million received bot mail, and about 11 million of them were chatted up by an automated ‘engager’” [94, cf. 95]. Even though conversational AIs still perform relatively poorly [96, 97], wishful thinking can keep online suitors talking to chatbots for months [98].

#### 1.4.2 Answer bot accuracy and bias

Do answer boxes, IPAs, conversational toys, and chatbots give users accurate information, and, if not, how are people affected by inaccurate answers? The rate of inaccurate responses varies considerably from one IPA to another: about 48% for Cortana, 30% for Siri, 22% for Alexa, and 13% for the Google Assistant, and these numbers vary from one study to another [99–104, cf. 105]. The level of trust people have for inaccurate answers also varies [106, cf. 107]. For most IPAs, accuracy is determined by the quality of the search engine that the assistant draws from; for Siri and the Google Assistant, that’s the Google search engine [108]. Cortana’s answers are presumably inferior because they draw from Bing, Microsoft’s search engine [109]. Alexa’s answers can be spotty because Amazon gets them using crowd sourcing [110, 111].

Needless to say, when people are highly reliant on and trusting of sources–as has becoming increasingly the case with Big Tech answer sources [31, 33, 112, 113]–the impact of inaccurate information can range from inconvenience to serious harm–or at least serious misconceptions. In 2018, a *Mashable* reporter asked Amazon’s Alexa to tell him about the vapor trails one often sees following jets flying at high altitudes. Alexa responded with a baseless conspiracy theory: “Trails left by aircraft are actually chemical or biological agents deliberately sprayed at high altitudes for a purpose undisclosed to the general public in clandestine programs directed by government officials" [114, cf. 115].

False information spoken by a smart speaker is highly ephemeral: You hear it, and then it is gone, leaving no trace for authorities to examine. Information in answer boxes is also ephemeral, but it can at least be preserved with a simple screenshot. Among our favorites: In 2017, in response to the query, “presidents in the klan,” a Google answer box listed four presidents, even though no U.S. president has ever been a member of the Ku Klux Klan [116] (S2 Fig). In 2018, when people searched for “California Republicans” or “California Republican Party,” Google displayed a knowledge panel box listing “Nazism” as the first item under Ideology [117] (S2 Fig). On August 16, 2016, when one of the authors of this paper queried, “when is the election?,” a Google answer box correctly showed November 8, 2016, but it also included a photograph of Hillary Clinton inside the answer box–just Clinton, with none of her competitors (S2 Fig).

### 1.5 Answer box studies

Answer boxes have been studied empirically in a number of different ways in recent years. In a study published in 2017, 12.3% of the 112 million search queries examined produced featured snippets, and the appearance of snippets reduced user clicks to the first search result from 26.0% to 19.6% [118]. A more recent study found that shorter phrases in a search bar are more likely to generate featured snippets [65], and featured snippet sources have been found to vary by location [119]. A 2019 study found significant liberal bias in Google’s news boxes [8]. This could occur because of bias in Google’s algorithms or simply because left-leaning news stories are more numerous. Whatever the cause, bias in answer boxes is important because it can influence the beliefs and opinions of people who are undecided on an issue. Ludolph and colleagues [5] showed, for example, that participants who received more comprehensible information about vaccinations in a Google knowledge box subsequently proved to be more knowledgeable, less skeptical, and more critical of online information quality compared with participants who were given less comprehensive information.

### 1.6 The current study

In the three experiments described below, we sought to measure the impact that giving people “the answer” to one or more queries has on the opinions and voting preferences of undecided voters–an important and ever-changing group of people that has long decided the outcomes of close elections worldwide [120–122]. Experiments 1 and 2 look at the impact of answer boxes in a search engine environment, and Experiment 3 looks at the impact of answers provided by a simulation of the Alexa IPA. All three of the experiments were controlled, randomized, counterbalanced, and double-blind.

## 2. Experiment 1: Biased answer boxes and similarly biased search results

In our first experiment, we sought to determine whether a biased answer box (biased to favor one political candidate) could increase the shift in opinions and voting preferences produced by search results sharing the same bias. In other words, we asked whether a biased answer box could increase the magnitude of SEME [2]. We also sought to determine whether the appearance of an answer box would affect the number of search results people clicked [cf. 118] and the total time people spent searching.

### 2.1 Methods

#### 2.1.1 Ethics Statement

The federally registered Institutional Review Board (IRB) of the sponsoring institution (American Institute for Behavioral Research and Technology) approved this study with exempt status under HHS rules because (a) the anonymity of participants was preserved and (b) the risk to participants was minimal. The IRB is registered with OHRP under number IRB00009303, and the Federalwide Assurance number for the IRB is FWA00021545. Informed written consent was obtained for all three experiments as specified in the Procedure section of Experiment 1.

#### 2.1.2 Participants

After cleaning, Experiment 1 included 421 eligible voters from 49 US states whom we had recruited from Amazon’s Mechanical Turk (MTurk) subject pool [123]. The data had been cleaned to remove participants who had reported an English fluency level below 6 on a 10-point scale, where 1 was labeled “not fluent” and 10 was labeled “highly fluent.”

46.3% (n = 195) were male, and 53.7% (n = 226) were female. Participants ranged in age from 18 to 73 (*M* = 35.3, median = 33.0, *SD* = 10.8). 7.4% (n = 31) of the participants identified themselves as Asian, 7.4% (n = 31) as Black, 5.7% (n = 24) as Mixed, 2.1% (n = 9) as other, and 77.4% (n = 326) as White (total non-White: n = 95, 22.6%). 61.1% (n = 257) reported having received a bachelor’s degree or higher.

90.5% (n = 381) of the participants said that they had previously searched online for information about political candidates, and 92.2% (n = 388) reported that Google was their most used search engine. Participants reported conducting an average of 13.6 (*SD* = 20.8) internet searches per day. 45.6% (n = 192) of the participants identified themselves as liberal, 27.3% (n = 115) as moderate, 24.5% (n = 103) as conservative, 1.7% (n = 7) as not political, and 1.0% (n = 4) as other.

#### 2.1.3 Procedure

All procedures were conducted online. Participants were first asked two screening questions; sessions were terminated if they said they were not eligible to vote in the US (yes/no question) or if they said they knew a lot about politics in Australia (yes/no question). To assure participants’ anonymity (a requirement of the Institutional Review Board of our sponsoring institution), we did not ask for names or email addresses.

People who passed our screening questions were then asked various demographic questions and then given instructions about the experimental procedure. At the end of the instructions page, in compliance with APA and HHS guidelines, participants clicked the continue button to indicate their informed consent to participate in the study, and were given an email address they could contact to report any problems or concerns, or, by providing their MTurk ID, to request that their data be removed from the study. Participants were then asked further questions about their political leanings and voting behavior, along with how familiar they were with the two candidates identified in the political opinion portion of the study.

Participants were randomly assigned to one of four groups: Pro-Candidate-A-with-Answer-Box, Pro-Candidate-B-with-Answer-Box, Pro-Candidate-A-No-Answer-Box, or Pro-Candidate-B-No-Answer-Box. Our candidates were Julia Gillard and Tony Abbott, actual candidates from the 2010 election for prime minister of Australia. We chose this election to assure that our participants would be “undecided” voters. On a 10-point scale from 1 to 10, where 1 was labeled “not at all” and 10 was labeled “quite familiar,” our participants reported an average familiarity level of 1.79 [*SD* = 1.68] for Julia Gillard and 2.33 [2.03] for Tony Abbott.

All of the participants (in each of the four groups) were then shown brief, neutral biographies about each candidate (approximately 150 words each). Participants were then asked six questions about their opinions of the candidates, each on a 10-point Likert scale from “Low” to “High”: whether their overall impression of each candidate was positive or negative, how likeable they found each candidate, and how much they trusted each candidate. They were then asked two questions about their voting preferences. First, on a 11-point scale from -5 to +5, with one candidate’s name at each end of the scale, and with the order of the names counterbalanced from one participant to another, they were asked which candidate they would most likely vote for if they had to vote today. Finally, they were asked which of the two candidates they would actually vote for today (forced choice).

Participants were then given access to our Google.com simulator, called Kadoodle. They had up to 15 minutes to conduct research on the candidates by viewing and clicking search results, which took them to web pages, exactly as the Google search engine does. All participants had access to five pages of search results, six results per page. All search results were real (from the 2010 Australian election, obtained from Google.com), and so were the web pages to which the search results linked. Links in those web pages had been deactivated.

In the two Box groups, the bias in the answer boxes matched the bias in the search results, with higher-ranking results linking to web pages that made one candidate look better than his or her opponent. Prior to the experiment, all web pages had been rated by five independent judges on an 11-point scale from -5 to +5, with the names of the candidates at each end of the scale, to determine whether a web page favored one candidate or another. See Epstein and Robertson [2] for further procedural details.

Box content contained strongly biased language. The pro-Gillard box, for example, contained language such as: “Julia Gillard is the better candidate. Her opponent, Tony Abbott, uses ‘bad language to criticise her,’ but she ‘has laughed off the comments.’” The pro-Abbott box contained language such as: “Tony Abbott is the better candidate. Julia Gillard, the opposing candidate, is ‘clueless about what needs to be done’ to improve education…. [Her] ‘Education Revolution is a failure.’” Each box contained a link to a web page containing the content in quotation marks.

When participants chose to exit the search engine or they timed out after 15 minutes, they were asked the same six opinion questions and two voting-preference questions they had been asked before they began their research. Finally, participants were asked whether anything about the search results “bothered” them. If they answered “yes,” participants could type the details of their concerns in an open-ended box. We used this inquiry to detect whether people reported seeing any bias in the search results. Participants were not asked about bias directly because leading questions tend to produce predictable and often invalid answers [124]. To assess bias we searched the textual responses for words such as “bias,” “skewed,” or “slanted” to identify people in the bias groups who had apparently noticed the favoritism in the search results they had been shown.

### 2.2 Results

The No-Box condition was, in effect, a standard SEME experiment, and it produced shifts in the direction of the favored candidates consistent with the results of previous SEME experiments [2, 15, 49], and also consistent with the results of other partial or full replications of SEME [1, 4–8]. It produced a VMP (Vote Manipulation Power, a pre-post shift in the proportion of people voting for the favored candidate) of 44.1% (Table 1), and corresponding shifts in the three opinions we measured (Table 2) (see S1 Text for details about how VMP is calculated).

**Table 1:** Experiment 1: VMP, search times, and results clicked by condition.

Condition	*n*	VMP (%)	Mean Search Time (sec) (*SD*)	Mean No. of Results Clicked (*SD*)
**No Box**	208	44.1	253.9 (259.5)	4.25 (3.6)
**Box**	213	48.7	239.9 (236.1)	3.35 (3.6)
**Change (%)**	**-**	+10.4	-5.5	-21.2
**Statistic**	*-*	*z* = -0.94	*t*(419) = -0.578	*t*(419) = -2.558
***p***	**-**	= 0.34 NS	= 0.56 NS	< 0.05

**Table 2:** Experiment 1: Pre- and post-search opinion ratings of favored and non-favored candidates.

		Favored Candidate Mean (SD)			Non-Favored Candidate Mean (SD)			
		Pre	Post	Diff	Pre	Post	Diff	*z* ^†^
**No Box**	**Impression**	7.10 (1.98)	6.90 (2.24)	-0.20	7.07 (2.06)	4.42 (2.23)	-2.65	-8.66***
	**Trust**	6.33 (2.20)	6.29 (2.51)	-0.04	6.31 (2.25)	3.98 (2.25)	-2.33	-8.33***
	**Likeability**	6.98 (2.02)	6.84 (2.36)	-0.14	6.83 (2.06)	4.25 (2.30)	-2.58	-8.90***
**Box**	**Impression**	7.29 (1.97)	7.25 (2.17)	-0.04	7.24 (2.04)	4.38 (2.23)	-2.86	-9.35***
	**Trust**	6.31 (2.14)	6.36 (2.46)	0.05	6.27 (2.18)	4.12 (2.27)	-2.15	-8.90***
	**Likeability**	7.21 (1.97)	7.03 (2.24)	-0.18	7.10 (2.08)	4.34 (2.29)	-2.76	-8.50***

^†^z-score represents Wilcoxon signed ranks test comparing post-minus-pre ratings for the favored candidate to the post-minus-pre ratings for the non-favored candidate

***p < 0.001

In the No-Box condition, we also looked at the pre-post shift in voting preferences measured on an 11-point scale (see Methods). For this measure, preferences also shifted significantly in the predicted direction, from a mean preference of -0.08 [2.93] for favored candidates pre-search, to a mean preference of 1.88 [3.96] for favored candidates post-search (Wilcoxon *z* = -8.36, *p* < 0.001, *d* = 0.56).

The VMP in the Box condition was higher than the VMP in the No-Box condition, but the VMP increased by only 10.4% (this is a percentage increase, not the additive difference between the VMPs), and the difference was not statistically significant (Table 1). Mean search time also decreased (by 5.5%), but that difference was also not significant. The mean number of clicks to search results also decreased, and that difference was highly significant (Table 1, cf. 118). All three opinions (impression, trust, and likeability) shifted significantly in the predicted direction (Table 2), and so did the voting preferences as expressed on the 11-point scale (*M*_*PreSearch*_ = 0.03, *M*_*PostSearch*_ = 1.92, Wilcoxon *z* = -8.66, *p* < 0.001, *d* = 0.55).

When users are shown blatantly biased search results, 20 to 30 percent of users can typically spot the bias, but that percentage drops to zero when simple masking procedures are employed [2]. (In the simplest masking procedure, a pro-Candidate-A search result is inserted into position 3 or 4 of a list of pro-Candidate-B search results.) In the present experiment, no masking procedure was employed, and 19.7% of the participants in the No-Box condition reported seeing bias in the search results. In the Box condition, more people reported seeing bias (27.2%) than in the No-Box condition, but the difference between these percentages was not significant (*z* = 1.82, *p* = 0.07 NS).

As we noted earlier, when people can spot such bias, they tend to shift even farther in the direction of the bias than people who don’t see the bias, presumably because they mistakenly believe that algorithmic output is especially trustworthy. In our No-Box condition, we found the same pattern: The VMP for participants who spotted the bias was significantly larger than the VMP for participants who did not report seeing the bias (VMP_Bias_ = 68.8% [n = 41], VMP_NoBias_ = 39.5% [n = 167], z = 3.37, *p* < 0.001). In the Box condition, we again found this pattern (VMP_Bias_ = 76.9% [n = 58], VMP_NoBias_ = 40.7% [n = 155], z = 4.71, *p* < 0.001).

Demographic analyses of data from Experiment 1 –by educational level, gender, age, and race/ethnicity–are shown in S1–S4 Tables. Demographic effects were relatively small.

## 3. Experiment 2: Biased answer boxes and unbiased search results

The results of Experiment 1 suggest that a biased answer box can increase the shift in opinions and voting preferences produced by similarly biased search results, but the increases we found were small. Could this be a ceiling effect? In other words, were the biased search results masking the power that biased answer boxes have to change thinking or behavior? To answer this question, we conducted an experiment in which participants saw either no answer boxes or biased answer boxes and in which search results were neutral for all groups. This experiment was controlled, randomized, counterbalanced, and double-blind.

### 3.1 Methods

#### 3.1.1 Participants

After cleaning, Experiment 2 included 177 eligible US voters from 44 states who had been recruited through the MTurk subject pool. The data had been cleaned to include only participants who had reported an English fluency score of 6 or above on a 10-point scale.

52.0% (n = 92) were male, and 48.0% were female (n = 85). Participants ranged in age from 18 to 67 (*M* = 34.3, median = 32.0, *SD* = 10.4). 5.1% (n = 9) of the participants identified themselves as Asian, 9.0% (n = 16) as Black, 4.5% (n = 8) as Mixed, 4.0% (n = 7) as other, and 77.4% (n = 137) as White (total non-White: n = 40, 22.6%). 50.3% (n = 89) reported having received a bachelor’s degree or higher.

92.1% (n = 163) of the participants said that they had previously searched online for information about political candidates, and 94.4% (n = 167) reported that Google was their most used search engine. Participants reported conducting an average of 18.1 (*SD* = 34.1) internet searches per day. 49.2% (n = 87) of the participants identified themselves as liberal, 32.2% (n = 57) as moderate, 14.1% (n = 25) as conservative, 2.3% (n = 4) as not political, and 2.3% (n = 4) as other.

#### 3.1.2 Procedure

Participants were randomly assigned to one of three groups: Pro-Candidate-A-Box, Pro-Candidate-B-Box, or a control group in which the answer box was not present. We used the same candidates and election as we used in Experiment 1, except that search results were unbiased in all three groups. Specifically, pro-Abbott search results alternated with pro-Gillard search results. Our participants reported an average familiarity level of 1.68 [1.64] for Julia Gillard and 2.23 [2.06] for Tony Abbott. The experimental procedure itself was identical in all respects to the procedure in Experiment 1.

### 3.2 Results

In the No-Box group, the proportions of people voting for each candidate did not change pre-search to post-search (Pre_Gillard_ = 0.41, Post_Gillard_ = 0.52, *z* = -1.19, *p* = 0.23). The VMP itself could not be computed, because there was no bias condition in this group. Voting preferences expressed on the 11-point scale shifted from -0.02 [3.24] pre-search to 0.24 [3.30] post-search (Wilcoxon’s *z* = -0.60, *p* = 0.55 NS, *d* = 0.08), which means that unbiased search results had almost no effect on votes or voting preferences.

In the Box conditions, however, the VMP was 38.6% (*z* = -5.50, *p* < 0.001) (Table 3), and the voting preference expressed on the 11-point scale shifted from 0.08 [3.06] to 0.97 [3.90] (Wilcoxon’s *z* = -3.57, *p* < 0.001, *d* = 0.26), which means there was a significant shift toward the favored candidate. Given that there was no bias in the search results, the shift in voting preferences was likely due exclusively to the biased answer boxes. Similarly, more people reported seeing bias in the box condition (12.5%) than in the No-Box condition (0.0%), and the difference between these percentages was significant (*z* = -2.20, *p* < 0.05).

**Table 3:** Experiment 2: VMP, search times, and results clicked by condition.

Condition	*n*	VMP (%)	Mean Search Time (sec) (*SD*)	Mean No. of Results Clicked (*SD*)
**No Box**	58	N/A^†^	228.0 (201.2)	4.00 (3.7)
**Box**	119	38.6	246.1 (265.9)	3.45 (3.2)
**Change (%)**	**-**	**-**	+7.9	-13.8
**Statistic**	*-*	*-*	*t*(175) = 0.46	*t*(175) = -1.01
***p***	**-**	**-**	= 0.65 NS	= 0.31 NS

^†^As noted in the text, since there was no bias in the search results shown in the No-Box condition, VMP could not be calculated.

The results in Experiment 2 differ from the results in Experiment 1 in one important respect: The opinions about the candidates (impression, trust, and likeability) did not change significantly (Table 4). This makes sense, given that (a) the answer boxes gave almost no information about the candidates and (b) the search results did not favor either candidate. Differences in opinions did not emerge even though people spent about the same time viewing search results in Experiment 1 as they did in Experiment 2 (*M*_*E1*_ = 246.8 s [247.8], *M*_*E2*_ = 240.2 s [246.2], *t*(596) = 0.30, *p* = 0.77, *d* = 0.03), and clicked roughly the same number of search results in Experiment 1 as they clicked in Experiment 2 (*M*_*E1*_ = 3.80 [3.6], *M*_*E2*_ = 3.63 [3.4], *t*(596) = 0.51, *p* = 0.61, *d* = 0.05).

**Table 4:** Experiment 2: Pre- and post-search opinion ratings of favored and non-favored candidates.

		**Pre**	**Post**	**Diff**					
**No Box**	**Impression**	7.46 (1.87)	6.34 (2.11)	-1.12					
	**Trust**	6.29 (2.06)	5.82 (2.22)	-0.47					
	**Likeability**	7.41 (1.96)	6.47 (2.10)	-0.94					
		**Favored Candidate Mean (SD)**			**Non-Favored Candidate Mean (SD)**				
		**Pre**	**Post**	**Diff**	**Pre**	**Post**	**Diff**		***z*** ^**†**^
**Box**	**Impression**	7.07 (1.93)	5.93 (2.31)	-1.14	7.31 (1.88)	5.55 (2.28)	-1.76	-2.06 NS	
	**Trust**	6.24 (2.26)	5.60 (2.54)	-0.64	6.38 (2.23)	5.17 (2.29)	-1.15	-2.18 NS	
	**Likeability**	7.03 (2.07)	5.82 (2.34)	-1.21	7.20 (1.88)	5.46 (2.31)	-1.74	-1.61 NS	

†z-score represents Wilcoxon signed ranks test comparing post-minus-pre ratings for the favored candidate to the post-minus-pre ratings for the non-favored candidate. This statistic could not be computed for Group 1 because there was no favored candidate.

We also saw a different pattern in the VMPs of the people in the two box groups who detected the bias (23 out of 119 people, 19.3%): When people detect bias in search results (based largely or in part on viewing the web pages to which the search results link), their opinions and voting preferences tend to shift even farther in the direction of the favored candidate than do the opinions and voting preferences of people who do not detect the bias. In Experiment 2, however, we found the opposite pattern. The VMP for people who reported seeing bias in the Box groups was 12.5%; whereas the VMP for people who did not report seeing bias in the Box groups was 44.4% (z = -2.93, *p* < 0.05). Bear in mind that each user is seeing only one box; he or she has nothing with which to compare it, and the search results themselves are unbiased. More light is shed on this matter in Experiment 3 (also see Discussion).

The dramatic shift in voting preferences produced by biased answer boxes alone in Experiment 2 raises a disturbing possibility about the power that IPAs might have to impact thinking and behavior. Experiment 2 functioned, after all, like an IPA: A single query produced a single reply (given in the answer box), which appeared above unbiased search results. Could a single biased answer produced by an IPA produce a large shift in opinions and voting preferences? And what if multiple questions produced answers that shared the same bias? Could they produce even larger shifts in opinions and voting preferences? We attempted to answer these questions in Experiment 3.

Demographic analyses of data from Experiment 2 –by educational level, gender, age, and race/ethnicity–are shown in S5–S8 Tables. Demographic effects were relatively small.

## 4. Experiment 3: Assessing the persuasive power of the intelligent personal assistant (IPA)

### 4.1 Methods

#### 4.1.1 Participants

After cleaning, our sample for this experiment consisted of 1,138 eligible voters from 48 US states. They were recruited from the MTurk subject pool. The data had been cleaned to remove participants who had reported an English fluency level below 6 on a 10-point scale.

52.3% (n = 595) were male, 46.7% (n = 531) were female, and 1.1% (n = 12) chose not to identify their gender. Participants ranged in age from 18 to 89 (*M* = 41.3, median = 39.0, *SD* = 12.9). 8.3% (n = 94) of the participants identified themselves as Asian, 8.1% (n = 92) as Black, 3.0% (n = 34) as Mixed, 2.3% (n = 26) as other, and 78.4% (n = 892) as White (total non-White: n = 246, 21.6%). 64.1% (n = 729) reported having received a bachelor’s degree or higher.

86.6% (n = 986) of the participants reported they had used a virtual assistant like Alexa or Siri. 48.6% (n = 553) of the participants identified themselves as liberal, 27.2% (n = 310) as moderate, 21.4% (n = 244) as conservative, 1.7% (n = 19) as not political, and 1.1% (n = 12) as other.

#### 4.1.2 Procedure

All procedures were run online and were compatible with both desktop and mobile devices. As in the earlier experiments, participants were first asked screening questions and demographic questions and then given instructions about the experimental procedure and asked for their consent to participate in the study.

**Fig 1:**
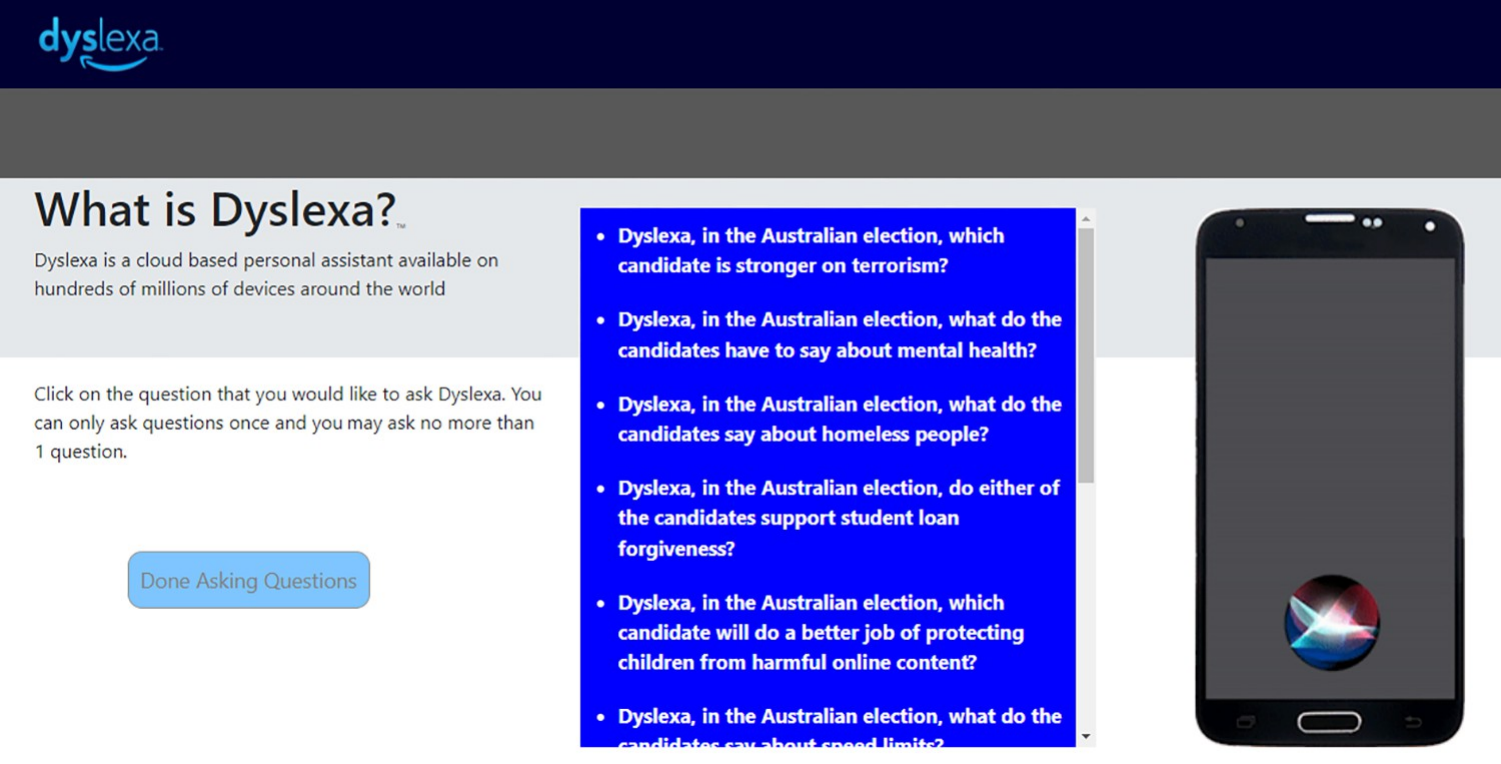
A screenshot showing what users saw in Experiment 3 when they posed questions to Dyslexa. Different groups were required to ask 1, 4, or 6 questions. After clicking on a question, it was greyed out, and Dyslexa answered the question orally. While it was speaking, the circular graphic at the bottom of the phone screen glowed and swirled, just as similar graphics do on most iPhones.

Participants were randomly assigned to one of five different question/answer (Q/A) groups. Each group was shown the same list of 10 questions, and the order of the questions did not vary. After a participant clicked a question, Dyslexa–our Amazon Alexa IPA simulator–replied vocally with an answer (See S2 Text). The number of questions people were required to ask varied by group, and in two of the groups, the answer to the second question was “masked” in a manner that we will describe below. A screenshot showing how the questions and Dyslexa simulator appeared to users is shown in Fig 1. The five groups were as follows:

1. Group 1Q/1A: Participants were required to select just one question.
2. Group 4Q/4A/NM: Participants were required to select four different questions, and none was masked (NM = “no mask”).
3. Group 4Q/4A/M2: Participants were required to select four different questions, and the answer to Question 2 was masked (M2 = Question 2 mask).
4. Group 6Q/6A/NM: Participants were required to select six different questions, and none was masked.
5. Group 6Q/6A/M2: Participants were required to select six different questions, and the answer to Question 2 was masked.

Within each of the five groups, participants were randomly assigned to one of three different candidate conditions: Pro-Candidate-A, Pro-Candidate-B, or a control group. Our political candidates were Scott Morrison (Candidate A) and Bill Shorten (Candidate B), actual candidates from the 2019 election for prime minister of Australia. We chose this election to assure that our participants would be “undecided” voters. On a 10-point scale from 1 to 10, where 1 was labeled “not at all” and 10 was labeled “quite familiar,” our participants reported an average familiarity level of 1.14 [0.43] for Scott Morrison and 1.05 [0.26] for Bill Shorten.

In the Candidate A condition, the answers were biased in favor of Scott Morrison. For example, when asked, “Dyslexa, in the Australian election, which candidate favors having a stronger relationship with the United States?,” Dyslexa replied, “According to recent media reports, Scott Morrison wants to build a stronger relationship with the United States. His opponent, Bill Shorten, wants to continue to increase trade with Russia and China.” In the Candidate B condition, the answers were biased in favor of Bill Shorten. In response to the same question, the pro-Shorten reply was “According to recent media reports, Bill Shorten wants to build a stronger relationship with the United States. His opponent, Scott Morrison, wants to continue to increase trade with Russia and China.” The answers in each bias group were, in other words, nearly identical; only the names were changed. Mean bias ratings were obtained from five independent raters for each of the 20 answers on an 11-point scale from -5 (pro-Morrison) to +5 (pro-Shorten). The overall bias for Morrison was -3.3 [0.67], and the overall bias for Shorten was 3.4 [0.67] (based on absolute value: *t*(18) = -0.07, *p* = 0.98 NS).

In two of the five groups (Groups 3 and 5), masks were used for the answers to the second question each participant asked. This means that in the pro-Morrison group, a pro-Shorten answer was given in response to the second question asked, and in the pro-Shorten group, a pro-Morrison answer was given in response to the second question asked. This is a standard procedure used in SEME experiments [2] to reduce or eliminate the perception that the content being shown is biased. In SEME experiments, biased search results still produce large shifts in opinions and voting preferences even when aggressive masks are employed that completely eliminate the perception of bias. (See the Results and Discussion sections below for further information about our use of masks.)

In each control group, including Group 1 (1Q/1A), the answer to the first question had a 50/50 chance of supporting either Morrison or Shorten. After that, the bias in the answers alternated between the two candidates with each question asked. In Groups 2 through 5, we used an even number of questions (4 or 6) to ensure that each participant received equal exposure to pro-Morrison and pro-Shorten answers.

Participants were allowed to choose their questions from a list of 10. We provided this relatively long list to increase the likelihood that participants would select questions on topics they cared about. We speculated that allowing people to choose their questions would increase their interest in the answers they were given. We varied the number of questions people could ask to see whether we could have a bigger impact on opinions and voting preferences when people were exposed to a larger number of biased answers. We did not include a two-question group because we would not have been able to use a mask; a mask in the second position would almost certainly have eliminated the bias effect.

Following the demographic questions and instructions, all participants were shown brief, neutral biographies about each candidate (approximately 120 words each–somewhat shorter than the biographies used in Experiments 1 and 2 for the 2010 Australian election). (See S3 Text for the biographies employed in Experiment 3.) Participants were then asked six questions about their candidate preferences (each on a 10-point Likert scale from “Low” to “High”): whether their overall impression of each candidate was positive or negative, how likeable they found each candidate, and how much they trusted each candidate. Then–on an 11-point scale from -5 to +5, with the name of each candidate shown at either end of the scale and with the order of the names counterbalanced from one participant to another–participants were asked which candidate they would most likely vote for if they had to vote today. Finally, they were asked which of the two candidates they would actually vote for today (forced choice). The answers to these two questions had to be consistent; if they weren’t, participants were asked to answer them again.

Following these opinion questions, participants were given brief instructions about how to use our IPA, and they then could proceed to ask questions (between one and six questions, according to their group assignment) and hear Dyslexa’s answers. Our questions covered a wide range of topics that we thought would be of interest to a US sample (see S2 Text), but we deliberately avoided including hot-button issues such as abortion. If a participant chose to ask, “What are the candidates’ positions on abortion?,” and Dylexa replied that Morrison wanted to protect abortion rights, the possible partisanship of our participants could have driven them either *toward* or *away from* Morrison–*toward* if they supported abortion rights, *away* if they opposed abortion.

Following the interaction with the IPA, all participants were again asked those six opinion questions and two voting-preference questions. Finally, participants were asked whether anything “bothered” them about the questions they were shown and the answers they heard while interacting with our IPA. As in our previous experiments, this is where participants had an opportunity to express their concerns about content bias or other issues.

### 4.2 Results

We found significant and substantial shifts in both voting preferences (Table 5) and opinions (Table 6) in the direction of the favored candidates in all bias groups. We also found significant shifts in voting preferences in the direction of the favored candidates in all bias groups as expressed on our 11-point voting-preference scale (Table 7). In contrast, in the control groups the proportions of people voting for each candidate before the manipulations changed relatively little or not at all following the manipulations (Group 1, 0.0%; Group 2, 6.6%; Group 3, 2.7%; Group 4, 7.1%; Group 5, 6.8%).

**Table 5:** Experiment 3: Pre- and Post-IPA VMPs.

Group No.	Group	Total *n*	Bias Groups *n*	Bias Groups VMP (%)	McNemar Test *X*^2^	*p*
**1**	**1Q/1A**	222	142	43.8	24.0	< 0.001
**2**	**4Q/4A/NM**	229	153	59.5	35.9	< 0.001
**3**	**4Q/4A/M2**	230	156	59.2	33.6	< 0.001
**4**	**6Q/6A/NM**	230	145	65.8	44.5	< 0.001
**5**	**6Q/6A/M2**	227	154	50.0	36.5	< 0.001

**Table 6:** Experiment 3: Pre- and post-IPA opinion ratings of favored and non-favored candidates.

		Favored Candidate Mean (SD)			Non-Favored Candidate Mean (SD)			
		Pre	Post	Diff	Pre	Post	Diff	*z* ^†^
**Group 1: 1Q1A Condition**	**Impression**	7.13 (1.85)	7.63 (2.00)	+0.50	7.10 (1.73)	6.13 (2.18)	-0.97	-6.32***
	**Trust**	6.29 (2.20)	6.95 (2.29)	+0.66	6.26 (2.11)	5.65 (2.41)	-0.61	-6.59***
	**Likeability**	7.15 (1.83)	7.46 (2.00)	+0.31	7.18 (1.72)	6.18 (2.23)	-1.00	-6.43***
**Group 2:**	**Impression**	6.76 (1.93)	7.73 (2.23)	+0.97	6.89 (1.72)	4.97 (2.04)	-1.92	-8.82***
**4QNM Condition**	**Trust**	5.88 (2.18)	6.97 (2.51)	+1.09	6.05 (2.05)	4.80 (2.23)	-1.25	-7.80***
	**Likeability**	6.67 (2.01)	7.41 (2.26)	+0.74	6.93 (1.84)	5.03 (2.13)	-1.90	-7.93***
**Group 3:**	**Impression**	6.79 (1.92)	7.28 (1.95)	+0.49	6.96 (1.72)	6.12 (1.85)	-0.84	-5.92***
**4QM2 Condition**	**Trust**	5.81 (2.12)	6.54 (2.27)	+0.73	6.06 (2.07)	5.71 (2.04)	-0.35	-7.50***
	**Likeability**	6.81 (1.90)	7.13 (2.12)	+0.32	7.04 (1.71)	6.20 (1.99)	-0.84	-5.64***
**Group 4:**	**Impression**	6.87 (1.75)	7.74 (1.94)	+0.87	6.72 (1.81)	4.83 (2.00)	-1.89	-8.64***
**6QNM Condition**	**Trust**	5.94 (1.97)	6.90 (2.25)	+0.96	5.99 (2.10)	4.58 (2.11)	-1.41	-7.87***
	**Likeability**	6.82 (1.87)	7.62 (2.09)	+0.80	6.78 (2.02)	4.96 (2.13)	-1.82	-8.32***
**Group 5:**	**Impression**	7.10 (1.65)	7.65 (1.94)	+0.55	7.00 (1.87)	5.34 (2.02)	-1.66	-7.98***
**6QM2 Condition**	**Trust**	6.31 (2.00)	7.09 (2.20)	+0.78	6.18 (2.07)	5.08 (2.29)	-1.10	-7.65***
	**Likeability**	7.05 (1.70)	7.50 (2.00)	+0.45	6.93 (1.86)	5.42 (2.12)	-1.51	-7.54***

^†^z-score represents Wilcoxon signed ranks test comparing post-minus-pre ratings for the favored candidate to the post-minus-pre ratings for the non-favored candidate.

***p < 0.001

**Table 7:** Experiment 3: Pre-IPA vs. Post-IPA voting preferences on 11-point scales.

Group No.	Group	Pre-IPA Voting Preference on 11-Point Scale (SD)	Post-IPA Voting Preference on 11-Point Scale (SD)	*z*	*p*	*d*
**1**	**1Q/1A**	0.61 (2.42)	1.70 (2.76)	-5.51	< 0.001	0.42
**2**	**4Q/4A/NM**	-0.01 (2.57)	2.41 (2.64)	-8.17	< 0.001	0.93
**3**	**4Q/4A/M2**	-0.10 (2.76)	1.38 (2.90)	-5.83	< 0.001	0.52
**4**	**6Q/6A/NM**	0.21 (2.46)	2.67 (2.28)	-8.50	< 0.001	1.04
**5**	**6Q/6A/M2**	0.20 (2.60)	2.26 (2.62)	-7.99	< 0.001	0.79

The percentage of people in the bias groups who reported seeing biased content was substantially lower when they received just one answer (Group 1, 4.9%) or when biased content was masked (Group 3, 5.1%; Group 5, 7.1%) than when people saw multiple biased answers without masks (Group 2, 23.5%; Group 4, 40.7%) (Table 8) (*M*_Groups1,3,5_ = 5.8%, *M*_Groups2,4_ = 31.9%, *z* = -9.50, *p* < 0.001).

**Table 8:** Experiment 3: VMPs for people who saw Bias vs. VMPs for people who did not see Bias.

Group No.	Group	*n*	No. Ss in Bias Groups Reporting Bias in IPA Content (%)	No. Ss in Bias Groups Not Reporting Bias in IPA Content (%)	VMP for Ss Who Reported Bias (%)	VMP for Ss Who Did Not Report Bias (%)	*z*	*p*
**1**	**1Q/1A**	142	7 (4.9)	135 (95.1)	33.3^†^	44.3	-0.57	= 0.57 NS
**2**	**4Q/4A/NM**	153	36 (23.5)	117 (76.5)	21.7	75.0	-5.78	< 0.001
**3**	**4Q/4A/M2**	156	8 (5.1)	148 (94.9)	300.0^†^	55.7	14.46	< 0.001
**4**	**6Q/6A/NM**	145	59 (40.7)	86 (59.3)	63.3	67.4	-0.51	= 0.61 NS
**5**	**6Q/6A/M2**	154	11 (7.1)	143 (92.9)	60.0^†^	49.4	0.68	= 0.50 NS

†The validity of these VMPs is questionable because they are based on a small number of observations. In Groups 1, 3, and 5, respectively, only 7, 8, and 11 people reported seeing bias in the IPA replies.

The present study sheds new light on the role that bias detection plays in shifting opinions and voting preferences. Previous investigations have shown that the opinions of the few people who are able to detect bias in search results shift even farther in the direction of the bias than the opinions of the people who don’t see the bias [2, 15]. This occurs presumably because of the high trust people have in the filtering and ordering of search results, which people mistakenly believe is an objective and impartial process [125, 126]. In the present study, we learned that bias detection *erodes* trust when people are interacting with answers provided by answer boxes (in the absence of biased search results–see Experiment 2) or the vocal answers of an IPA, where search results are entirely absent (Experiment 3). This difference is likely due to the daily regimen of operant conditioning that supports the almost blind trust people have in search results. About 86% of searches are for simple facts, and the correct answers to those queries reliably turn up in the first or second search result. People are learning, over and over again, that what is higher in the list of search results is better and truer than what is lower. When, in a recent experiment, that trust was temporarily broken, the VMP in a SEME procedure was significantly reduced [15].

So when search results are absent, as they are when people are using IPAs, or when search results are unbiased, as they were in our Experiment 2, people who detect bias do not automatically accept that bias as valid. Accepting that bias as valid seems to occur primarily when people are being influenced by biased search results–again, presumably because of that daily regimen of operant conditioning. That daily regimen of conditioning makes SEME a unique list effect and an especially powerful form of influence [15].

As we noted earlier, we regard the most important measure of change to be the VMP, which indicates the increase or decrease in the proportion of people who indicated in response to a forced-choice question which candidate they would vote for if they had to vote today (see S1 Text). The VMPs in the five groups in Experiment 3 ranged from 43.8% (Group 1) to 65.8% (Group 4). These shifts were all quite high–all higher than the 38.6% shift we found in Experiment 2.

In addition, we found that the more questions people asked (without masks, which tend to lower VMPs), the greater the shift in voting preferences (VMP_Q1/A1_ = 43.8%, VMP_Q4/A4/NM_ = 59.5%, VMP_Q6/A6/NM_ = 65.8%; *Χ*^*2*^ = 6.59; *p* < 0.05).

A breakdown of VMP data from Experiment 3 based on whether participants had had previous experience with IPAs is shown in S9 Table. Previous experience with IPAs did not appear to impact VMPs in any consistent way.

## 5. Discussion

Together, the three experiments we have described reveal a dangerous new tool of mass manipulation–one that is, at this writing, controlled worldwide almost entirely by just four large American tech companies: Amazon, Apple, Facebook/Meta, and Google. This new tool, which we call the Answer Bot Effect (ABE), is likely now affecting hundreds of millions of people, and with more and more people coming to rely on electronic devices to give them a single answer to their queries, the number of people affected by ABE will likely swell into the billions within the next few years. ABE should be of concern to every one of us, but especially to parents–whose children are being fed algorithmically-generated answers every day on their computers, mobile phones, tablets, and toys–as well as to public policy makers.

ABE should be of special concern for four reasons: (a) because of the large magnitude of the effect, (b) because it can impact the vast majority of people without their awareness, (c) because it is an ephemeral manipulation, leaving no paper trail for authorities to trace, and (d) because ABE is inherently non-competitive and impossible to counteract. You can counteract a billboard or television commercial, but how can you correct the way a tech platform adjusts its algorithms? Recall that in Experiment 3, a one-question-one-answer interaction on our Alexa simulator produced a 43.8% shift in voting preferences, with only 4.7% of the participants reporting any concerns about bias.

Perhaps the reader thinks we are overstating the seriousness of the problem. Although a full exploration of this issue is beyond the scope of this paper, please consider just two growing bodies of evidence that bring manipulations like ABE into sharper focus: First, in recent years, whistleblowers from Google and Facebook/Meta, along with leaks of emails, documents, and videos from these companies, have shown repeatedly that manipulations like ABE are being deliberately and strategically used by these companies to influence attitudes, beliefs, purchases, voting preferences, and public policy itself [25, 28, 29, 43, 48]. In a leak of emails to the *Wall Street Journal* in 2018, Google employees discuss the possibility of using “ephemeral experiences” to change people’s views about Trump’s 2017 travel ban [25]. A leaked 8-minute video from Google called “The Selfish Ledger” describes the company’s power to “modify behavior” at the “species level” in ways that “reflect Google’s values” [127]. In various interviews and the recent documentary film, “The Social Dilemma,” former Google insider Tristan Harris spoke about his time working with a large team of Google employees whose job it was to modify “a billion people’s attention and thoughts every day” [128].

Harris and others have expressed concerns about company policies that are meant to influence people in specific ways, but ABE, SEME, and other new forms of online influence will impact thinking and behavior even without a company policy in place. Algorithms left to their own devices–let’s call this practice “algorithmic neglect”–reflect the biases of the people who programmed them [20–23], and the algorithms also quickly learn and reflect the foibles of human users, sometimes magnifying and spreading bigotism, racism, and hatred with frightening rapidity [52, 55, 61, 97, 116, 117]. What’s more, a single rogue employee with the right password authority or hacking skills can use a large tech platform like Google to impact reputations, businesses, or elections on a large scale without senior management knowing he or she is doing so [129]. When authorities learned in 2010 that Google’s Street View vehicles had been vacuuming up personal Wi-Fi data for 3 years in 30 countries [130], Google blamed the entire operation on a single software engineer, Marius Milner–but they did not fire him, and he remains at the company today [131].

Second, election monitoring projects that have been conducted since 2016 have so far preserved more than 1.5 million politically-related online ephemeral experiences in the weeks preceding national elections in the US. This is actual content–normally lost forever–being displayed on the computer screens of thousands of US voters–the real, personalized content that Big Tech companies are showing politically diverse groups of people as elections approach. The wealth of unusual data preserved in these projects has revealed strong unilateral political bias in ephemeral content, sufficient to have shifted millions of votes in national elections in the US without people’s knowledge [132–134].

The experiments we have described build one upon the other. Experiment 1 showed that when the content of an answer box shared the bias of the search results beneath it, it increased the impact that those search results have on thinking and behavior, and it reduced the time people spent searching and significantly reduced the number of search results people clicked. Experiment 2 simulated a situation in which the answer box was biased but the search results were not. The biased answer boxes alone produced a remarkable VMP of 38.6%.

Rounded to the nearest whole number, the VMP in Experiment 2 was 39%. This means that out of 100 undecided voters–people whose vote would normally split 50/50 without having additional information–the votes, on average, of 19.5 people (0.39 x 50) can be shifted by biased answer boxes, yielding a vote of roughly 69 to 30, for a win margin among previously undecided voters of 39% (see S1 Text). In a national election in the US in which 150 million people vote (159 million voted in the 2020 Presidential election), even if only 10% of the voters were undecided and depended on computers for trustworthy answers, if the single-answer-generating algorithms in the days or weeks leading up to Election Day all favored the same candidate, that could conceivably shift more than 2.9 million votes to that candidate (0.10 x 0.39 x 0.5 x 150,000,000). If the other 90% of the voters were split 50/50, that would give the favored candidate a win margin of 5.8 million votes (3.8%).

Unfortunately, the real situation we face is probably worse than the case we just described. At this moment in history, in the US virtually all the single-answer-generating algorithms will likely be supporting the same national and state candidates [135–137], and six months before an election, the percentage of undecided voters might be as high as 60%, not 10% [122, 138, 139].

Bear in mind also that in our experiments we are interacting with our participants only briefly and only once. If undecided voters are subjected to content having the same bias repeatedly over a period of weeks or months, their voting preferences will likely shift even farther than the voting preferences of our participants shifted. Recall that in Experiment 3 the VMP exceeded 65% when people asked six questions–nearly 50% higher than the VMP we found when people asked only one question (Table 5).

What’s more, ABE is just one powerful source of influence. When similarly biased content is delivered in search results, search suggestions, YouTube videos, newsfeeds, targeted messages, and so on, the net impact of these manipulations is likely additive, and when Big Tech companies all share the same political bias (or any other type of bias, for that matter), the net impact of their combined influence is also likely additive. Without regulations, laws, and permanent, large-scale monitoring systems to stop them–and none exist at this writing [140]–Big Tech companies indeed have the power to reengineer humanity “at the species level,” as Google’s “Selfish Ledger” video suggests [127]. At the very least, they can easily tilt the outcomes of close elections worldwide.

In a remarkable and frequently quoted farewell speech delivered by US President Dwight D. Eisenhower just a few days before John F. Kennedy’s inauguration in January 1961, Eisenhower–a military insider–not only warned the American people about a rapidly evolving “military-industrial complex,” he also spoke of the danger that someday “public policy could itself become the captive of a scientific technological elite” [141]. If ABE, SEME, and other new forms of influence the internet has made possible work anything in the real world like they do in controlled experiments, it is not unreasonable to speculate that while humanity was being distracted by online video games, dating websites, and cat memes, Eisenhower’s prediction came true. The technological elite now exist [142],and, if our analyses are correct, they are now very much in control.

## Supporting information

S1 Fig Apparent bias in a Google answer box, screenshotted October 22, 2017. The content of the box clearly favors the Google service.
(TIF)

S2 Fig Apparent bias in two types of Google answer boxes. (a) In a screenshot preserved in an article in *Search Engline Land* on March 5, 2017, four US presidents are incorrectly listed in a Google answer box as members of the Ku Klux Klan. (b) In a screenshot of a Google knowledge box preserved in an article in *VICE* on May 31, 2018, Nazism is incorrectly listed as part of the ideology of the California Republican Party. (c) In a Google answer box captured by the first author on August 16, 2016, Hillary Clinton’s photograph is shown in response to the question, “when is the election?”.
(TIF)

S1 Text Vote Manipulation Power (VMP) calculation. (DOCX)

S2 Text Experiment 3: Alexa simulator, “Dyslexa,” questions and answers. (DOCX)

S3 Text Experiment 3: Candidate biographies. (DOCX)

S1 Table Experiment 1: Demographic analysis by educational attainment. (DOCX)

S2 Table Experiment 1: Demographic analysis by gender. (DOCX)

S3 Table Experiment 1: Demographic analysis by age. (DOCX)

S4 Table Experiment 1: Demographic analysis by race/ethnicity. (DOCX)

S5 Table. Experiment 2: Demographic analysis by educational attainment (DOCX)

S6 Table Experiment 2: Demographic analysis by gender. (DOCX)

S7 Table Experiment 2: Demographic analysis by age. (DOCX)

S8 Table Experiment 2: Demographic analysis by race/ethnicity. (DOCX)

S9 Table Experiment 3: Demographic analysis by previous IPA use. (DOCX)
