# Supplementary figures and images for: The Answer Bot Effect (ABE): A powerful new form of influence made possible by intelligent personal assistants and search engines
Source: PLoS One. 2022 Jun 1;17(6):e0268081. doi: 10.1371/journal.pone.0268081 (PMC9159602; doi:10.1371/journal.pone.0268081)

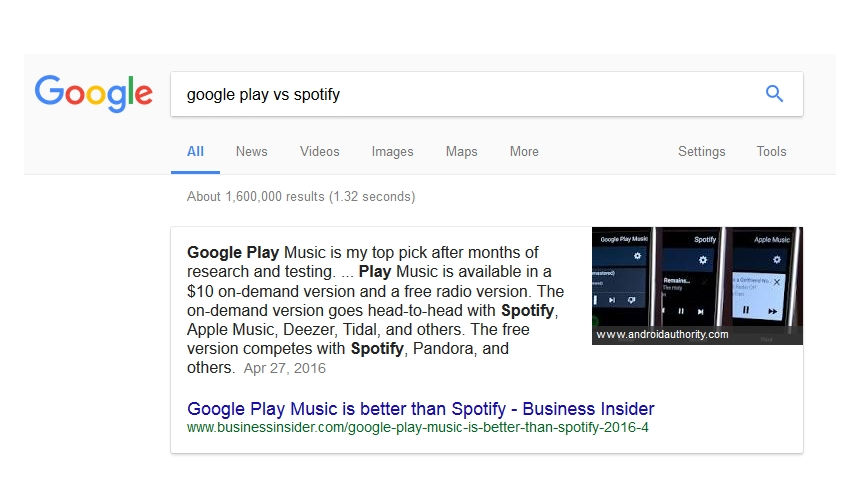

Supplement: S1 Fig — The content of the box clearly favors the Google service. (TIF) [file pone.0268081.s001.tif]

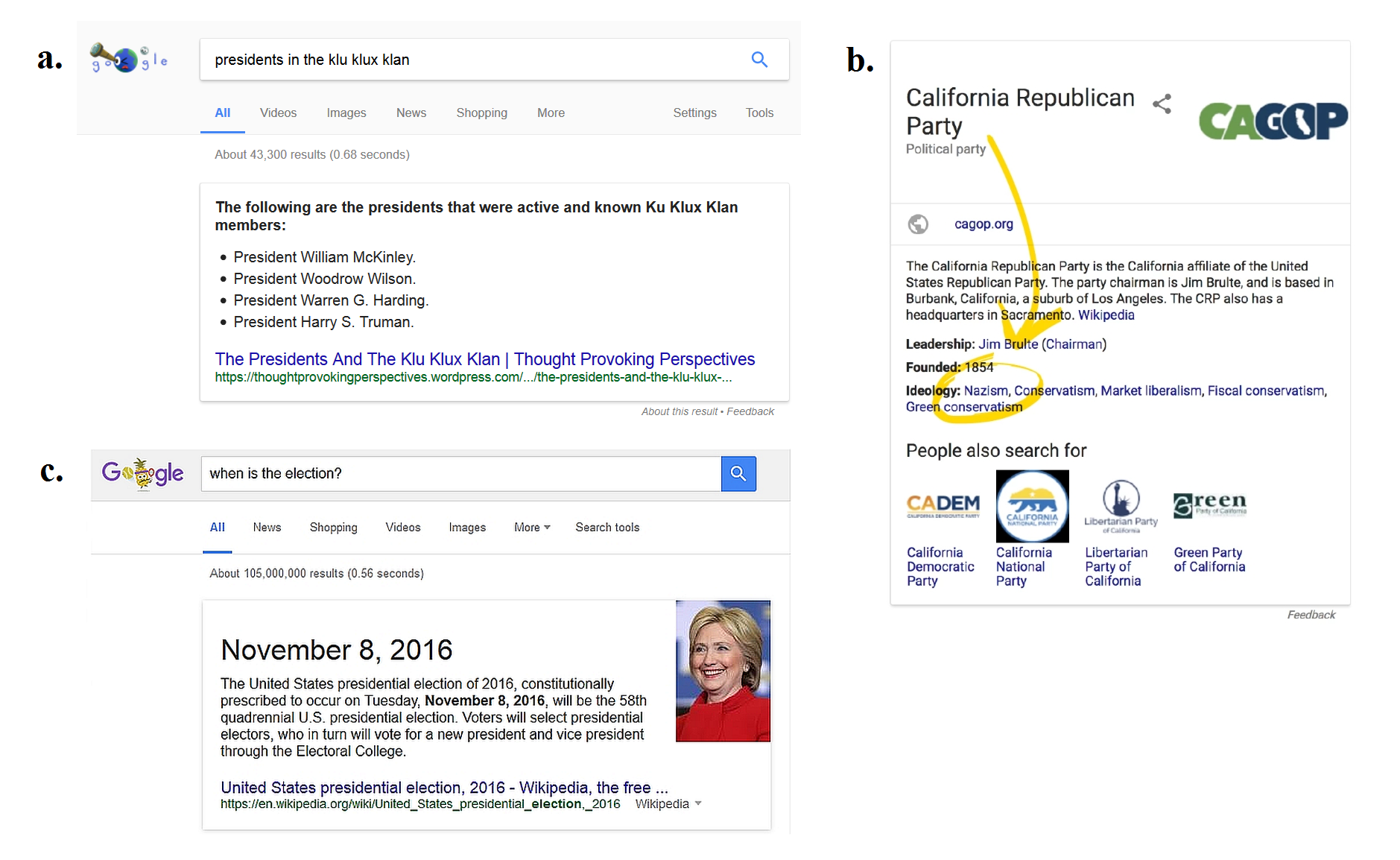

Supplement: S2 Fig — (a) In a screenshot preserved in an article in Search Engline Land on March 5, 2017, four US presidents are incorrectly listed in a Google answer box as members of the Ku Klux Klan. (b) In a screenshot of a Google knowledge box preserved in an article in VICE on May 31, 2018, Nazism is incorrectly listed as part of the ideology of the California Republican Party. (c) In a Google answer box captured by the first author on August 16, 2016, Hillary Clinton’s photograph is shown in response to the question, “when is the election?”. (TIF) [file pone.0268081.s002.tif]
