# Supplementary material for: The Answer Bot Effect (ABE): A powerful new form of influence made possible by intelligent personal assistants and search engines
Source: PLoS One. 2022 Jun 1;17(6):e0268081. doi: 10.1371/journal.pone.0268081 (PMC9159602; doi:10.1371/journal.pone.0268081)
Supplement: S1 Text — (DOCX) [file pone.0268081.s003.docx]

**S1 Text. Vote Manipulation Power (VMP) calculation.**

Vote Manipulation Power (VMP) is calculated as follows:

*p' – p*
 *p*

where *p* is the total number of people who voted for the favored candidate pre-manipulation, and *p'* is the total number of people who voted for the favored candidate post-manipulation. If, pre-manipulation, a group of 100 people is split 50/50 in the votes they give us, and if, post-manipulation, a total of 67 people now vote for the favored candidate, the VMP is

*67 – 50*
 *50*

or 34%. Because *p'* is 17 points larger than *p*, the win margin is 34 (2 x 17, or 34%), and the final vote is 67 to 33, with the favored candidate the winner. So in any group in which the vote is split 50/50 pre-manipulation, the VMP is also the win margin. Note that 17 individuals did not need to *shift* to produce this win margin. We only needed the *net* number of people voting for the favored candidate to be 67.
