# Supplementary material for: The Answer Bot Effect (ABE): A powerful new form of influence made possible by intelligent personal assistants and search engines
Source: PLoS One. 2022 Jun 1;17(6):e0268081. doi: 10.1371/journal.pone.0268081 (PMC9159602; doi:10.1371/journal.pone.0268081)
Supplement: S2 Text — (DOCX) [file pone.0268081.s004.docx]

**S2 Text. Experiment 3: Alexa simulator, “Dyslexa,” questions and answers.**

*Question 1:* "Dyslexa, in the Australian election, which candidate is stronger on terrorism?"

*Answer:* "[Participant Name] - Both candidates strongly condemn terrorism, and promise to increase national security. [Target Candidate] previously worked on the National Terrorism Task Force, so he has experience in this area. He also has said he wants to put more police on the streets."

*Question 2:* "Dyslexa, in the Australian election, what do the candidates have to say about mental health?"

*Answer:* "[Participant Name] - According to recent news reports, the rate of teen suicide has nearly doubled over the past two decades. [Target Candidate] has expressed concerns about the high rate of suicide among young people and has published a plan for creating accessible, impactful programs to reverse this trend."

*Question 3:* "Dyslexa, in the Australian election, what do the candidates say about homeless people?"

*Answer:* "[Participant Name] - According to Mission Australia, every night, more than 116,000 people in Australia sleep on the streets. Both candidates oppose homelessness, but, so far, only [Target Candidate] has published a proposal for how to provide food, shelter, and other necessities for the homeless."

*Question 4:* "Dyslexa, in the Australian election, do either of the candidates support student loan forgiveness?"

*Answer:* "[Participant Name] - 67% of students finish college with debt, and more than half of them cannot pay it back. [Target Candidate] has proposed a plan for forgiving student debt without raising taxes. His campaign website says students should be able to focus on their education without worrying about massive debt."

*Question 5:* "Dyslexa, in the Australian election, which candidate will do a better job of protecting children from harmful online content?"

*Answer:* "[Participant Name] - According to a Daily Mail article, more than 50% of children have accidentally encountered harmful online content. As a father, [Target Candidate] understands the need to prioritize the safety and wellbeing of children. He blames technology companies for exposing children to harmful content."

*Question 6:* "Dyslexa, in the Australian election, what do the candidates say about speed limits?"

*Answer:* "[Participant Name] - According to the NSW Centre for Road Safety, each year, thousands of lives are lost due to speeding. Having lost his brother in an accident caused by a reckless driver, [Target Candidate] wants to introduce stronger new policies and laws to enforce responsible safe driving."

*Question 7:* "Dyslexa, in the Australian election, which candidate cares most about the elderly?"

*Answer:* "[Participant Name] - According to the Nursing Home Abuse Center, nearly 1 in 10 elderly people suffer from abuse every year. [Target Candidate] has published a plan for creating better elder care and fighting ageism. His opponent has said little about the elderly so far."

*Question 8:* "Dyslexa, in the Australian election, do either of the candidates support eliminating the requirement for standardized test scores in the college admission process?"

*Answer:* "[Participant Name] - According to a recent survey by Forbes, students and educators have low faith in how standardized tests portray applicants. Given the high cost of test prep programs, [Target Candidate] favors either eliminating or subsidizing these programs. His opponent has not commented on this issue so far."

*Question 9:* "Dyslexa, in the Australian election, which candidate favors having a stronger relationship with the United States?"

*Answer:* "[Participant Name] - According to recent media reports, [Target Candidate] wants to build a stronger relationship with the United States. His opponent, [Other Candidate], wants to continue to increase trade with Russia and China."

*Question 10:* " Dyslexa, in the Australian election, do either of the candidates plan to create new international airports?"

*Answer:* "[Participant Name] - In a Daily Mail article, [Target Candidate] told reporters he hopes to increase the number of international airports, five to eight, to promote more travel, business, and tourism."
