# Supplementary material for: The Answer Bot Effect (ABE): A powerful new form of influence made possible by intelligent personal assistants and search engines
Source: PLoS One. 2022 Jun 1;17(6):e0268081. doi: 10.1371/journal.pone.0268081 (PMC9159602; doi:10.1371/journal.pone.0268081)
Supplement: S1 Table — (DOCX) [file pone.0268081.s006.docx]

**S1 Table. Experiment 1: Demographic analysis by educational attainment.**

| **Condition** |  | ***n*** | **VMP (%)** | **Mean Search Time (sec) (SD)** | **Mean No. of Results Clicked (SD)** |
| --- | --- | --- | --- | --- | --- |
| **No Box** | **≥ Bachelors** | 130 | 46.0 | 243.6 (248.6) | 4.18 (3.2) |
|  | **< Bachelors** | 77 | 38.5 | 274.4 (277.7) | 4.40 (4.1) |
|  | **Change (%)** | - | -16.3 | +12.6 | +5.3 |
|  | **Statistic** | *-* | *z* = 1.05 | t(205) = 0.82 | t(205) = 0.44 |
|  | ***p*** | - | = 0.29 NS | = 0.41 NS | = 0.66 NS |
| **Box** | **≥ Bachelors** | 127 | 58.8 | 225.3 (224.5) | 3.46 (3.9) |
|  | **< Bachelors** | 86 | 34.7 | 261.4 (252.2) | 3.19 (3.2) |
|  | **Change (%)** | - | -41.0 | +16.0 | -7.8 |
|  | **Statistic** | *-* | *z* = 3.45 | t(211) = 1.10 | *t*(211) = -0.55 |
|  | ***p*** | - | < 0.001 | = 0.28 NS | = 0.59 NS |
