# Supplementary material for: The Answer Bot Effect (ABE): A powerful new form of influence made possible by intelligent personal assistants and search engines
Source: PLoS One. 2022 Jun 1;17(6):e0268081. doi: 10.1371/journal.pone.0268081 (PMC9159602; doi:10.1371/journal.pone.0268081)
Supplement: S2 Table — (DOCX) [file pone.0268081.s007.docx]

**S2 Table. Experiment 1: Demographic analysis by gender.**

| **Condition** |  | ***n*** | **VMP (%)** | **Mean Search Time (sec) (SD)** | **Mean No. of Results Clicked (SD)** |
| --- | --- | --- | --- | --- | --- |
| **No Box** | **Male** | 103 | 38.5 | 231.7 (258.4) | 4.0 (3.7) |
|  | **Female** | 105 | 55.6 | 275.6 (260.1) | 4.5 (3.4) |
|  | **Change (%)** | - | +44.4 | +18.9 | +12.5 |
|  | **Statistic** | *-* | *z* = -2.28 | t(206) = -1.22 | t(206) = -1.04 |
|  | ***p*** | - | < 0.05 | = 0.22 NS | = 0.30 NS |
| **Box** | **Male** | 92 | 34.7 | 214.4 (222.0) | 3.6 (4.2) |
|  | **Female** | 121 | 58.3 | 259.3 (245.5) | 3.1 (3.1) |
|  | **Change (%)** | - | +68.0 | +20.9 | -13.9 |
|  | **Statistic** | *-* | *z* = -3.35 | t(211) = -1.38 | *t*(211) = 1.01 |
|  | ***p*** | - | < 0.001 | = 0.17 NS | = 0.31 NS |
