# Supplementary material for: The Answer Bot Effect (ABE): A powerful new form of influence made possible by intelligent personal assistants and search engines
Source: PLoS One. 2022 Jun 1;17(6):e0268081. doi: 10.1371/journal.pone.0268081 (PMC9159602; doi:10.1371/journal.pone.0268081)
Supplement: S3 Table — (DOCX) [file pone.0268081.s008.docx]

**S3 Table. Experiment 1: Demographic analysis by age.**

| **Condition** |  | ***n*** | **VMP (%)** | **Mean Search Time (sec) (SD)** | **Mean No. of Results Clicked (SD)** |
| --- | --- | --- | --- | --- | --- |
| **No Box** | **≥ 33** | 106 | 74.4 | 308.4 (279.7) | 4.8 (3.5) |
|  | **< 33** | 102 | 22.0 | 197.2 (224.5) | 3.7 (3.5) |
|  | **Change (%)** | - | -70.4 | -36.1 | -22.9 |
|  | **Statistic** | *-* | *z* = 7.56 | t(200) = -3.17 | t(206) = -2.26 |
|  | ***p*** | - | < 0.001 | < 0.01 | < 0.05 |
| **Box** | **≥ 33** | 113 | 65.5 | 283.0 (252.5) | 3.6 (3.4) |
|  | **< 33** | 100 | 33.9 | 191.2 (206.7) | 3.0 (3.9) |
|  | **Change (%)** | - | -48.2 | -32.4 | -16.7 |
|  | **Statistic** | *-* | *z* = 4.60 | t(210) = -2.92 | *t*(211) = -1.22 |
|  | ***p*** | - | < 0.001 | < 0.01 | = 0.23 NS |
