# Supplementary material for: The Answer Bot Effect (ABE): A powerful new form of influence made possible by intelligent personal assistants and search engines
Source: PLoS One. 2022 Jun 1;17(6):e0268081. doi: 10.1371/journal.pone.0268081 (PMC9159602; doi:10.1371/journal.pone.0268081)
Supplement: S4 Table — (DOCX) [file pone.0268081.s009.docx]

**S4 Table. Experiment 1: Demographic analysis by race/ethnicity.**

| **Condition** |  | ***n*** | **VMP (%)** | **Mean Search Time (sec) (SD)** | **Mean No. of Results Clicked (SD)** |
| --- | --- | --- | --- | --- | --- |
| **No Box** | **White** | 159 | 54.9 | 253.9 (262.0) | 4.2 (3.3) |
|  | **Non-White** | 49 | 19.4 | 253.7 (254.2) | 4.5 (4.4) |
|  | **Change (%)** | - | -64.7 | -0.1 | +7.1 |
|  | **Statistic** | *-* | *z* = 4.36 | t(206) = 0.01 | t(206) = -0.54 |
|  | ***p*** | - | < 0.001 | = 0.10 NS | = 0.59 NS |
| **Box** | **White** | 167 | 47.3 | 239.9 (235.4) | 3.1 (3.0) |
|  | **Non-White** | 46 | 53.8 | 239.8 (241.5) | 4.4 (5.4) |
|  | **Change (%)** | - | +13.7 | -0.0 | +41.9 |
|  | **Statistic** | *-* | *z* = -0.78 | t(211) = 0.00 | *t*(53) = -1.61 |
|  | ***p*** | - | = 0.44 NS | = 1.00 NS | = 0.11 NS |
