# Supplementary material for: The Answer Bot Effect (ABE): A powerful new form of influence made possible by intelligent personal assistants and search engines
Source: PLoS One. 2022 Jun 1;17(6):e0268081. doi: 10.1371/journal.pone.0268081 (PMC9159602; doi:10.1371/journal.pone.0268081)
Supplement: S5 Table. Experiment 2: Demographic analysis by educational attainment — (DOCX) [file pone.0268081.s010.docx]

**S5 Table. Experiment 2: Demographic analysis by educational attainment.**

| **Condition** |  | ***n*** | **VMP (%)** | **Mean Search Time (sec) (SD)** | **Mean No. of Results Clicked (SD)** |
| --- | --- | --- | --- | --- | --- |
| **No Box** | **≥ Bachelors** | 29 | N/A^†^ | 269.9 (229.5) | 4.8 (4.5) |
|  | **< Bachelors** | 28 | N/A^†^ | 191.3 (161.9) | 3.3 (2.4) |
|  | **Change (%)** | - | - | -29.1 | -31.3 |
|  | **Statistic** | *-* | *-* | t(55) = 1.49 | t(44) = 1.67 |
|  | ***p*** | - | - | = 0.14 NS | = 0.10 NS |
| **Box** | **≥ Bachelors** | 60 | 45.8 | 245.8 (300.7) | 3.2 (2.9) |
|  | **< Bachelors** | 59 | 30.0 | 246.5 (227.9) | 3.8 (3.5) |
|  | **Change (%)** | - | -34.5 | +0.3 | +18.8 |
|  | **Statistic** | *-* | *z* = 1.78 | t(117) = -0.02 | *t*(117) = -1.04 |
|  | ***p*** | - | = 0.08 NS | = 0.99 NS | = 0.30 NS |

^†^As noted in the text, since there was no bias in the search results shown in the No-Box condition, VMP could not be calculated.
