# Supplementary material for: The Answer Bot Effect (ABE): A powerful new form of influence made possible by intelligent personal assistants and search engines
Source: PLoS One. 2022 Jun 1;17(6):e0268081. doi: 10.1371/journal.pone.0268081 (PMC9159602; doi:10.1371/journal.pone.0268081)
Supplement: S6 Table — (DOCX) [file pone.0268081.s011.docx]

**S6 Table. Experiment 2: Demographic analysis by gender.**

| **Condition** |  | ***n*** | **VMP (%)** | **Mean Search Time (sec) (SD)** | **Mean No. of Results Clicked (SD)** |
| --- | --- | --- | --- | --- | --- |
| **No Box** | **Male** | 27 | N/A^†^ | 226.9 (218.1) | 3.8 (3.9) |
|  | **Female** | 54 | N/A | 228.9 (188.8) | 4.2 (3.5) |
|  | **Change (%)** | - | - | +0.9 | +10.5 |
|  | **Statistic** | *-* | *-* | t(56) = -0.04 | t(56) = -0.36 |
|  | ***p*** | - | - | = 0.97 NS | = 0.72 NS |
| **Box** | **Male** | 65 | 25.9 | 203.2 (266.5) | 3.1 (2.8) |
|  | **Female** | 31 | 58.8 | 297.7 (258.3) | 3.9 (3.6) |
|  | **Change (%)** | - | +123.9 | +46.5 | +25.8 |
|  | **Statistic** | *-* | *z* = -3.64 | t(117) = -1.95 | *t*(117) = -1.46 |
|  | ***p*** | - | < 0.001 | = 0.05 NS | = 0.15 NS |

^†^As noted in the text, since there was no bias in the search results shown in the No-Box condition, VMP could not be calculated.
