# Supplementary material for: The Answer Bot Effect (ABE): A powerful new form of influence made possible by intelligent personal assistants and search engines
Source: PLoS One. 2022 Jun 1;17(6):e0268081. doi: 10.1371/journal.pone.0268081 (PMC9159602; doi:10.1371/journal.pone.0268081)
Supplement: S7 Table — (DOCX) [file pone.0268081.s012.docx]

**S7 Table. Experiment 2: Demographic analysis by age.**

| **Condition** |  | ***n*** | **VMP (%)** | **Mean Search Time (sec) (SD)** | **Mean No. of Results Clicked (SD)** |
| --- | --- | --- | --- | --- | --- |
| **No Box** | **≥ 32** | 33 | N/A^†^ | 247.6 (207.0) | 4.0 (3.7) |
|  | **< 32** | 25 | N/A^†^ | 202.1 (194.3) | 4.0 (3.7) |
|  | **Change (%)** | - | - | -18.4 | +0.0 |
|  | **Statistic** | *-* | *-* | t(56) = -0.85 | t(56) = 0.00 |
|  | ***p*** | - | - | = 0.40 NS | = 0.65 NS |
| **Box** | **≥ 32** | 58 | 30.4 | 301.2 (318.3) | 3.7 (4.0) |
|  | **< 32** | 61 | 47.6 | 193.7 (192.7) | 3.2 (2.4) |
|  | **Change (%)** | - | +56.6 | -35.7 | -13.5 |
|  | **Statistic** | *-* | *z* = -1.92 | t(93) = -2.24 | *t*(93) = -0.77 |
|  | ***p*** | - | = 0.05 NS | < 0.05 | = 0.45 NS |

^†^As noted in the text, since there was no bias in the search results shown in the No-Box condition, VMP could not be calculated.
