# Supplementary material for: The Answer Bot Effect (ABE): A powerful new form of influence made possible by intelligent personal assistants and search engines
Source: PLoS One. 2022 Jun 1;17(6):e0268081. doi: 10.1371/journal.pone.0268081 (PMC9159602; doi:10.1371/journal.pone.0268081)
Supplement: S8 Table — (DOCX) [file pone.0268081.s013.docx]

**S8 Table. Experiment 2: Demographic analysis by race/ethnicity.**

| **Condition** |  | ***n*** | **VMP (%)** | **Mean Search Time (sec) (SD)** | **Mean No. of Results Clicked (SD)** |
| --- | --- | --- | --- | --- | --- |
| **No Box** | **White** | 47 | N/A^†^ | 216.2 (204.9) | 3.8 (3.6) |
|  | **Non-White** | 11 | N/A^†^ | 278.2 (184.8) | 4.9 (4.8) |
|  | **Change (%)** | - | - | -28.7 | +28.9 |
|  | **Statistic** | *-* | *-* | t(56) = 0.92 | t(56) = 0.92 |
|  | ***p*** | - | - | = 0.36 NS | = 0.36 NS |
| **Box** | **White** | 90 | 39.4 | 246.9 (248.3) | 3.5 (3.4) |
|  | **Non-White** | 29 | 36.4 | 243.7 (319.6) | 3.4 (2.6) |
|  | **Change (%)** | - | -7.6 | -1.3 | -2.9 |
|  | **Statistic** | *-* | *z* = 0.29 | t(117) = -0.06 | *t*(117) = -0.08 |
|  | ***p*** | - | = 0.77 NS | = 0.96 NS | = 0.94 NS |

^†^As noted in the text, since there was no bias in the search results shown in the No-Box condition, VMP could not be calculated.
