# Supplementary material for: The Answer Bot Effect (ABE): A powerful new form of influence made possible by intelligent personal assistants and search engines
Source: PLoS One. 2022 Jun 1;17(6):e0268081. doi: 10.1371/journal.pone.0268081 (PMC9159602; doi:10.1371/journal.pone.0268081)
Supplement: S9 Table — (DOCX) [file pone.0268081.s014.docx]

**S9 Table. Experiment 3: Demographic analysis by previous IPA use.**

| **Group No.** | **Group** | **Have used IPA** | | **Have not used IPA** | |  |  |  |
| --- | --- | --- | --- | --- | --- | --- | --- | --- |
|  |  | **VMP (%)** | ***n*** | **VMP (%)** | ***n*** | **Diff (%)** | ***z*** | ***p*** |
| **1** | **1Q/1A** | 37.3 | 123 | 116.7 | 19 | +79.4 | -6.45 | < 0.001 |
| **2** | **4Q/4A/NM** | 59.4 | 131 | 60.0 | 22 | +0.60 | -0.05 | 0.96 |
| **3** | **4Q/4A/M2** | 58.5 | 141 | 66.7 | 15 | +8.2 | -0.61 | 0.54 |
| **4** | **6Q/6A/NM** | 72.3 | 128 | 27.3 | 17 | -45.0 | 3.71 | < 0.001 |
| **5** | **6Q/6a/M2** | 52.8 | 137 | 33.3 | 17 | -19.5 | 1.52 | 0.13 |
